# Evolutionary relationships between miRNA genes and their activity

**DOI:** 10.1186/1471-2164-13-718

**Published:** 2012-12-22

**Authors:** Yan Zhu, Geir Skogerbø, Qianqian Ning, Zhen Wang, Biqing Li, Shuang Yang, Hong Sun, Yixue Li

**Affiliations:** 1 Key Laboratory of Systems Biology, Shanghai Institutes for Biological Sciences, Chinese Academy of Sciences, Shanghai, 200031, China; 2 Shanghai Center for Bioinformation Technology, Shanghai, 200235, China; 3 Department of Cardiology, Gansu Provincial Hospital, Lanzhou, 730000, China; 4 School of Life Science and Technology, Tongji University, Shanghai, 200092, China

## Abstract

**Background:**

The emergence of vertebrates is characterized by a strong increase in miRNA families. MicroRNAs interact broadly with many transcripts, and the evolution of such a system is intriguing. However, evolutionary questions concerning the origin of miRNA genes and their subsequent evolution remain unexplained.

**Results:**

In order to systematically understand the evolutionary relationship between miRNAs gene and their function, we classified human known miRNAs into eight groups based on their evolutionary ages estimated by maximum parsimony method. New miRNA genes with new functional sequences accumulated more dynamically in vertebrates than that observed in Drosophila. Different levels of evolutionary selection were observed over miRNA gene sequences with different time of origin. Most genic miRNAs differ from their host genes in time of origin, there is no particular relationship between the age of a miRNA and the age of its host genes, genic miRNAs are mostly younger than the corresponding host genes. MicroRNAs originated over different time-scales are often predicted/verified to target the same or overlapping sets of genes, opening the possibility of substantial functional redundancy among miRNAs of different ages. Higher degree of tissue specificity and lower expression level was found in young miRNAs.

**Conclusions:**

Our data showed that compared with protein coding genes, miRNA genes are more dynamic in terms of emergence and decay. Evolution patterns are quite different between miRNAs of different ages. MicroRNAs activity is under tight control with well-regulated expression increased and targeting decreased over time. Our work calls attention to the study of miRNA activity with a consideration of their origin time.

## Background

MicroRNAs are small endogenously expressed single-stranded RNAs, that regulate gene expression post transcriptionally and shape diverse cellular pathways [1-3]. MicroRNA families have continuously been added to the vertebrate lineage, and when integrated into a genome, a miRNA gene is only rarely lost [4-6]. MicroRNAs date back to the earliest bilaterians, and specific miRNA families operating in specific cells and tissues of both primitive protostomes and primitive deuterostomes have been identified [7], suggesting very early metazoan origin [8]. The emergence of vertebrates is characterized by a strong increase in miRNA families, and correlates with the increase in vertebrate morphological complexity [6,9]. Almost all nodes within Metazoa are characterized by addition of miRNA families that are rarely lost in the descendants [10]. The miRNA family acquisition rate at the emergence of vertebrates have been estimated to 0.9-2.7 families per Myr, and many of these 41 miRNA families show tissue or cell type specific expression, miRNAs may thus lie at the basis of cell and tissue specification in vertebrates [11]. Acquisition of miRNA genes apparently speed up with evolution of organismal complexity.

MicroRNAs’ effects on target gene expression can be roughly classified into two types: “tuning” and “buffering”. Tuning relates to effects on the target gene expression level, whereas buffering relates to repression of expressional variation [12]. It is speculated that the dual functions of miRNAs could represent two stages in miRNA evolution, miRNA initially acting by reducing variance in gene expression, and only gradually taking on tuning of the expressional level over time [12]. Apparently, miRNAs of varying age are not “equal”, as older miRNAs are commonly more highly and broadly expressed than younger miRNAs [13], and knockout of an older miRNA results in a more severe phenotype than knockout of a younger miRNA [14,15]. Liang *et. al* divided miRNAs into groups based on their expression level, the sequence divergence in the mature regions of miRNAs with higher expression level is significantly lower than that in the remaining regions, and miRNAs with very low expression tend to turn over quickly in evolution [16]. It has been suggested that lowly expressed miRNAs may occasionally be selected and included into the regulatory network [13,17]. If newly emerged “miRNAs” find targets, their regulation would probably be detrimental [18], however, they may also serve as substrate for natural selection of beneficial target interactions [13,19,20], and newly activated miRNAs may be part of general mechanism by which speciation occurs [18].

Based on the observations that intraspecific variation decrease through evolutionary time, that miRNA decrease stochastic expressional variation, and that miRNA numbers increase through evolutionary time and with morphological complexity, it has been suggested that miRNA are at the basis of the canalization development required for increased organismal complexity [21]. Simulation of selection in presence or absence of miRNA regulation suggested that evolution of population did not take place in absence of miRNA genes [21]. By constraining the gene expression, miRNAs renders phenotypic traits governed by (spatiotemporal) gene expression more “heritable”, and thereby “evolvable” [22]. The evolution of miRNA system is intriguing, however, evolutionary questions concerning the origin of miRNA genes and their subsequent evolution remain unexplained. In order to systematically understand the evolutionary relationship between miRNAs gene and their function, in this work, we focused specifically on human miRNAs for their diversified activities during evolution.

## Results

### MicroRNA emergence and evolution

To further study the evolution of human miRNAs, we divided the human known miRNA genes (miRBase v.15) into eight age groups according to their time of origin as estimated by the maximum parsimony method [23] (Additional file 1: Figure S1). During the first 150 Myrs of vertebrate evolution, the lineage leading to human accumulated less new miRNA genes, compared to ~250 miRNA genes during the last 50 Myrs of evolution along the same lineage (Additional file 1: Figure S2A). In order to estimate the evolutionary turnover rates for human miRNA genes, we applied a method from Lu et al. [24]. This showed that the miRNA birth rate in vertebrates is more than 40 new miRNA genes per Myr, which is about three times higher than that observed in Drosophila (12/Myr; [24]). A large proportion of acquired miRNA genes degenerate rapidly (Additional file 1: Figure S2B), and only around five percent of new vertebrate miRNAs survived in the long run of evolution (100 Myrs). This is nonetheless twice the net increase in miRNA genes found in Drosophila, in which only 2.5% of surviving in Drosophila [24], and accumulation of new miRNA genes in vertebrates for most of their evolution (Additional file 1: Figure S2A).

The long-term net gain (= birth - death) in vertebrates is two miRNAs per Myr, which is more than six times that found in Drosophila [24]. Accumulation rates of extant miRNA were slow during the earlier stages of vertebrate evolution, and proceeded with increasing speed during the late stages. A large class of human miRNA genes are thus of very recent origin, 55% of all human miRNA genes having originated after the primate-rodent split. There is little overlap in mature or seed sequences between the age groups, that is, in most cases miRNAs within a given age group are distinct from miRNAs in other age groups (Table 1). The data suggests that most emerging miRNA genes produce new functional sequences. Unlike coding genes, miRNAs is a most dynamic class of genes in terms of emergence and decay.

**Table 1:** Number of miRNAs with same functional sequence between age groups

**A. Number of miRNAs with same mature sequence**									
**Age groups**	**t0**	**t1**	**t2**	**t3**	**t4**	**t5**	**t6**	**t7**	**Unique (%)**
t0	77	1	3	6	1	1	0	0	85
t1	1	60	4	5	1	1	0	0	97
t2	3	4	85	5	0	1	0	0	93
t3	6	5	5	244	0	2	0	0	89
t4	1	1	0	0	27	0	0	0	90
t5	1	1	1	2	0	250	2	2	98
t6	0	0	0	0	0	2	67	3	96
t7	0	0	0	0	0	2	3	61	92
**B. Number of miRNAs with same seed sequence**									
t0	76	3	6	9	1	5	1	1	84
t1	3	58	6	9	1	5	1	0	94
t2	6	6	82	14	0	4	1	0	90
t3	9	9	14	225	0	13	1	0	82
t4	1	1	0	0	27	0	0	0	90
t5	5	5	4	13	0	226	13	11	88
t6	1	1	1	1	0	13	61	4	87
t7	1	0	0	0	0	11	4	52	79

### Mutations were frequent in young miRNAs and decreased as miRNAs became fixed

We compared signatures of natural selection on human miRNAs with those of same-length flanking genomic sequences, assuming flanking regions to be selectively close to neutral [25]. We first studied the sequence variation in humans. The percentages of pre-miRNAs containing SNP(s) are much lower when compared with flanking regions, and decreases with increasing evolutionary age (Additional file 1: Figure S3A). For both miRNA precursors and mature sequences, the densities of SNPs are relatively higher for most of newly emerged miRNA genes than those from old groups. (Additional file 1: Figure S3B).

The degree of sequence conservation is very strong for the miRNA age groups that originated before the primate-rodent split (Additional file 1: Figure S3C), suggesting strong purifying selection on both precursors and mature sequences of old miRNA genes; whereas for young miRNAs the sequence substitution rates are rapid for both functional sequences (precursors and mature sequences) and their nearby flanking genomic regions, which are more than three times higher than that for old miRNAs (Additional file 1: Figure S3C). These results strongly indicated different levels of evolutionary selection over miRNA genes with different time of origin, for young miRNA genes weak (or no) selection are both observed in miRNA genes and their surrounding genomic regions.

### Difference in sequence and structure characteristics between miRNA age groups

**Figure 1:**
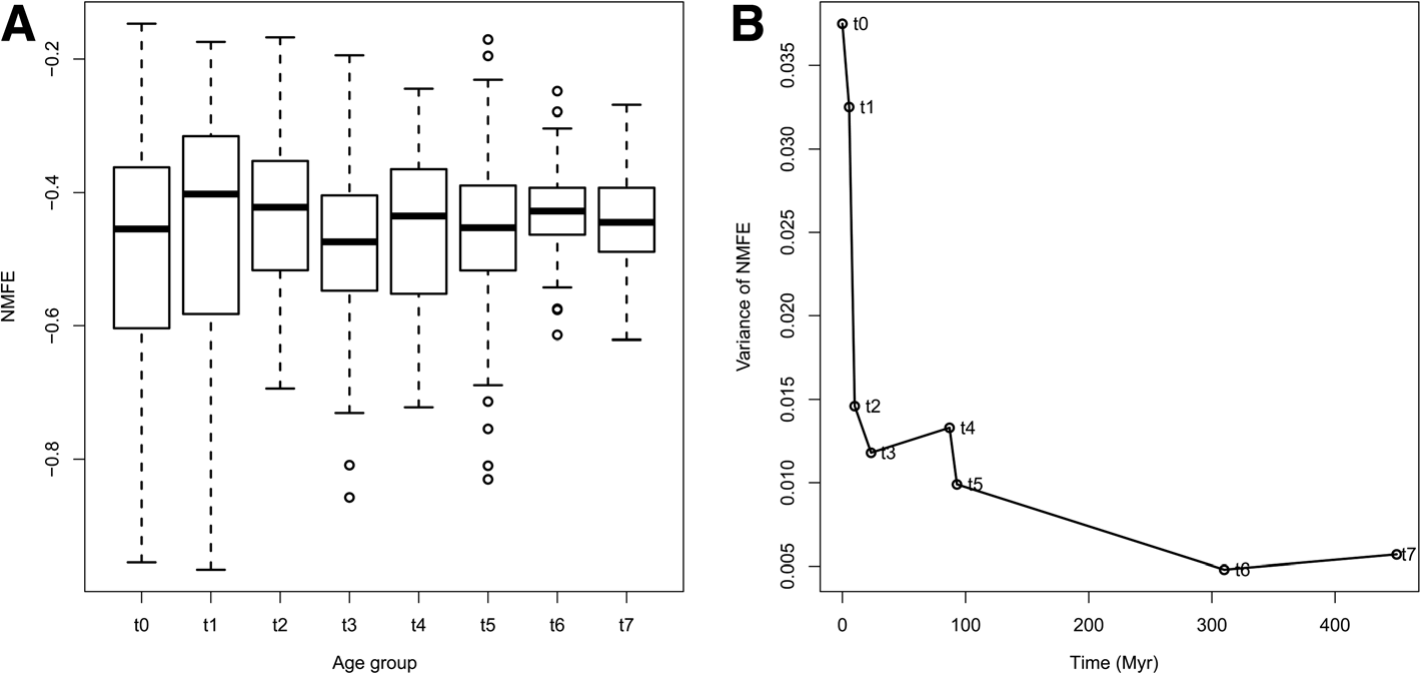
**Energetic properties of pre-miRNAs’ secondary structure for each age group.** Normalized minimum free energy (NMFE) (**A**) and NMFE variance (**B**).

We assessed various aspects of the miRNAs and their precursors across the eight age groups. The average normalized minimum free energy (NMFE) of the pre-miRNAs did not differ much between the age groups (Figure 1A), however, the NMFE variance was considerable lower for the oldest groups, and increased more than 6 fold from the oldest (t7) to the youngest (t0) group of miRNAs (Figure 1B). A recent study in Drosophila showed that the high robustness of pre-miRNAs secondary structure is likely to be a consequence of selection for functional structures [26]. The relatively rapid sequence evolution of young pre-miRNAs (Additional file 1: Figure S3) may have a influence on the robustness of their stem-loop structures, thus contributing to the NMFE variance. The much lower variance of NMFE observed in old pre-miRNAs indicated that old miRNAs may have undergone changes to acquire a sequence composition with optimized sequence and energetic properties necessary for successful recognition and processing by all parts of the miRNA-processing apparatus.

Further analysis of the relationship between miRNA evolutionary age and 48 features used for prediction of miRNA precursors [27] indicated that while the majority of these showed little systematic variation with miRNA age, several displayed a consistent increasing or decreasing trend with increasing miRNA age. Among these were several features related to structure and nucleotide composition (Additional file 1: Figure S4).

We also took advantage of a recently published miRNA prediction tool utilizing integrated sequence-structure motifs to identify miRNA precursors (ss-motifs [28]). Analysis of the miRNA precursor from the different age groups show the number of ss-motifs identified in each age group increased substantially with increasing miRNA age (Table 2). There are more miRNAs in age group t3 and t5, and the resulting less average number of ss-motifs indicated that there is a redundancy in ss-motifs among miRNAs, and that the number of ss-motifs does not increase linearly with the number of miRNAs. For each age group, a number of group-specific ss-motifs were identified, and these were also far more abundant in the older age groups (Table 2), suggesting that the older miRNA precursors have more sequence-structure in common than younger miRNA precursors. Comparison to the set ss-motifs identified as being most informative with respect to computational prediction of miRNA precursor (*i.e.,* “informative” ss-motifs [29]), showed that more “informative” ss-motifs were identified among older than younger miRNA precursors (Table 2).

**Table 2:** Average number of ss-motifs

	**t0**	**t1**	**t2**	**t3**	**t4**	**t5**	**t6**	**t7**
Number of miRNAs	91	62	91	273	30	256	70	66
Motif_LR	29.53	40.37	32.61	23.67	150. 67	23.77	131.06	197.95
Common to motif 1300	0.12	0.36	0.14	0.11	1.48	0.07	0.76	1.86
Specific motifs	0.13	0.87	0.17	0.62	23.93	0.41	17.84	68.23

Computational identification for pre-miRNA features depends on the knowledge of known miRNA genes, which may bring bias towards well-known miRNA genes (most are well conserved among species). In another perspective, precursors of old miRNAs have acquired a sequence composition for successful recognition and processing by all parts of the miRNA-processing apparatus over long-term evolution, and as a consequence of selection for functional structures, ss-motifs extracted from old pre-miRNAs may be more “informative” for feature analysis.

### Unbalanced evolution between intronic miRNAs and host genes

**Figure 2:**
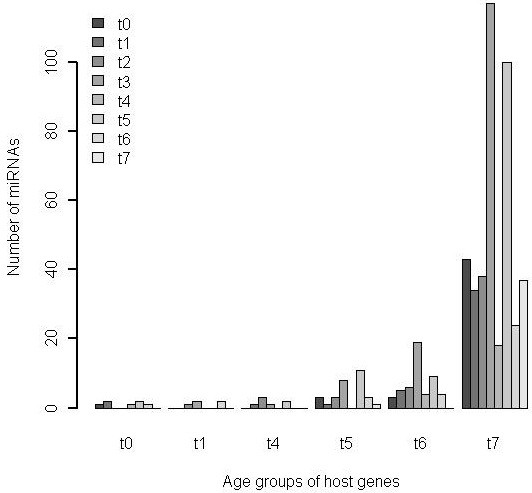
Unparallel origin of genic miRNAs and their host genes.

A considerable fraction (63%) of human miRNA genes have intronic loci. Several studies have suggested relationships between intronic miRNAs and the functional roles of their host genes [14,30-32] which may benefit from the expressional co-regulation of miRNA and host gene [33]. A total of 442 Ensembl genes host one or more miRNAs in sense and/or antisense strand. Gene Ontology analysis of these miRNA host genes revealed a broad spectrum of biological roles, classified into 58 functional clusters. However, no particular relationship was found between the age of a miRNA and the age of its host genes (Figure 2), nor did miRNA genes of different age vary systematically in their propensity to occupy intronic loci (Additional file 1: Table S1). The latter may suggests that there are selective pressures to maintain this kind of genomic organization pattern, or that intronic loci offer more chances for activation of novel miRNA genes than do most other noncoding sequence. This pattern of genomic localization may advantageous to the transcription of miRNAs of both emerging and established miRNAs [34].

Most genic miRNAs differ from their host genes in time of origin, and only around one-tenth shared the time period of origin with their host gene. Genic miRNAs are mostly younger than the corresponding host genes, and ancient coding genes host most of genic miRNAs (90%) (Figure 2, Additional file 1: Table S1). One most intriguing example is that three miRNA genes with distinct origin are encoded by the introns of AATYK gene (Additional file 1: Figure S5), hsa-mir-338 is highly conserved among vertebrates, hsa-mir-657 originated after the primate-rodent split, and hsa-mir-1250 originated after the human-chimp split. Previous studies have showed the capacity of has-mir-338 to modulate AATYK mRNA levels during the maturation of rat hippocampal neurons [35], and co-transcription together with the host gene silencing a family of mRNAs whose protein products are negative regulators of neuronal differentiation [36]. AATYK is an apoptosis-associated tyrosine kinase and specifically expressed in human brain, which is the most complex tissue in the human body, these three miRNAs with different time of origin could have a role in establishing or maintaining cellular diversity and could thereby contribute to the differences in human brain evolution and function.

### Synergistic targeting of multi-age miRNAs

**Figure 3:**
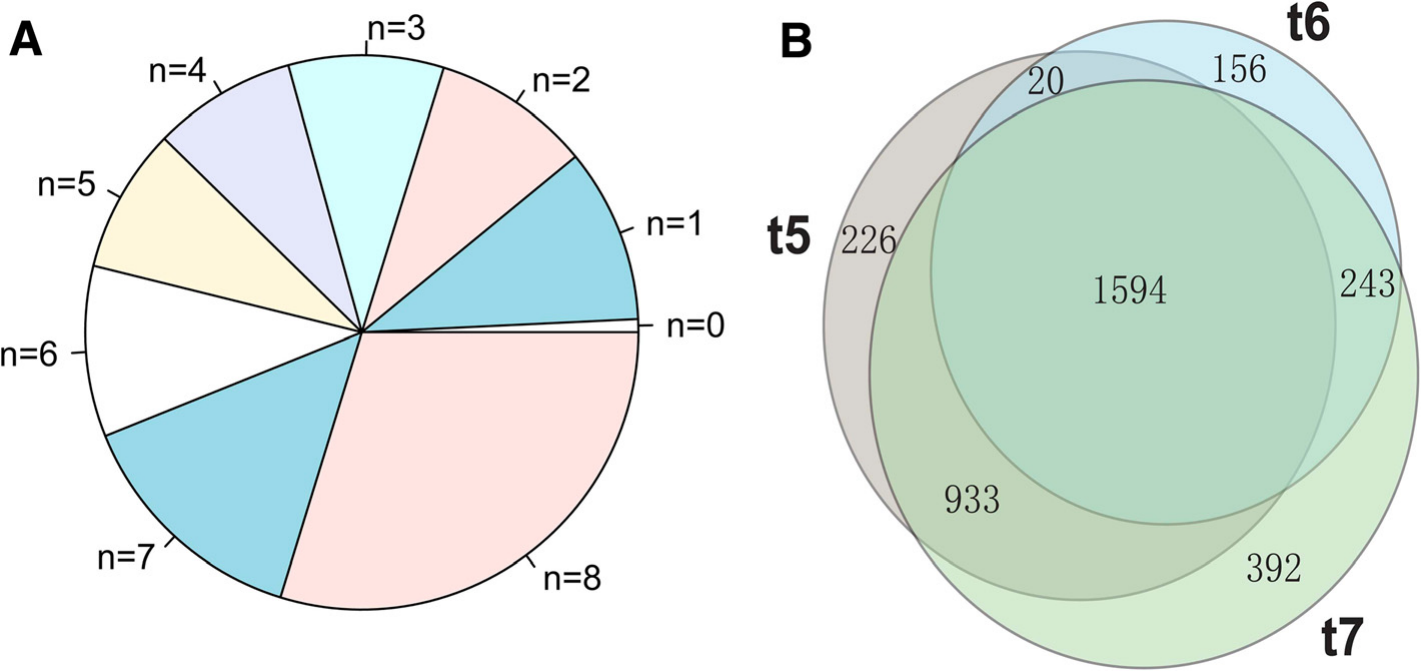
**Synergistic targeting of multi-age miRNAs.** Composition of coding genes with multiple paring sites for miRNAs of diversified origin times (**A**). Overlapping number of targets among miRNA age groups (**B**).

The average number of predicted targets with selectively maintained sites increase significantly with increasing of miRNA age (Additional file 1: Table S2), and the same tendency was observed for experimentally verified miRNA-target pairs [37] (Additional file 1: Table S3). Most of miRNA target genes have multiple miRNA binding sites (Figure 3A) as was also observed from the experimental data [37] (Figure 3B), and miRNAs from different age groups commonly target the same or overlapping sets of genes, thus opening the possibility of substantial functional redundancy among miRNAs of different ages.

### Difference in expression profiles between miRNA age groups

To characterize trends in expression along with the evolution of miRNAs, we first used a data set of 378 human pre-miRNAs expression atlas, each of which has tissue expression data in normal and malignant cells and tissues [38]. As small number of newly emerged miRNAs had expression data, the eight age groups were combined into three: those miRNAs originated after the primate-rodent split, the young group, which contains 87 miRNAs; those originated after rodent-non-mammalian split and before the primate-rodent split, the middle age group, which contains 174 miRNAs; and those originated before Bos taurus, the old age group, which contains 117 miNRAs. We calculated a specificity score to assess the tissue specificity of miRNA expression following the method used by Landgraf *et al.*[38]. The measure of miRNA transcript abundance used here is the maximal expression level among the small RNA libraries [16].

**Figure 4:**
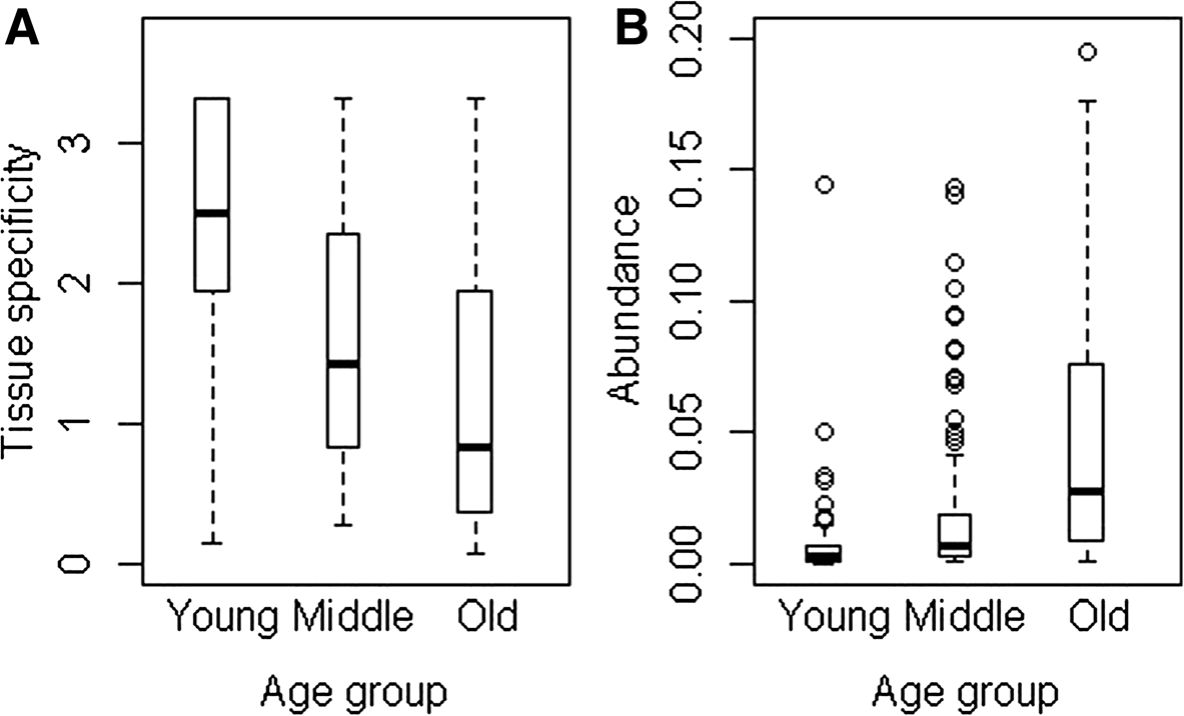
Expression of miRNA age groups, tissue specificity (A) and abundance (B).

The expression of young miRNAs is under tight control. The specificity scores decrease with miRNAs age, and the expression levels increase across tissues from all sample libraries (Figure 4 A,B). Compared with relatively old groups of miRNAs, the tissue specificity of young miRNAs is significantly higher and the expression levels are significantly lower (Table 3). The high degree of tissue specificity and low expression level of young miRNAs suggests a strict control on expression accounting for the young miRNAs activity and well-regulated expression increased over time.

**Table 3:** Abundance and tissue specificity of human known miRNAs’ expression

**Samples**		**Young**^**a**^	**Middle**^**a**^	**Old**^**a**^	**P value (Young,Middle)**	**P value (Young,Old)**	**P value (Middle,Old)**
Tissue specificity score (median)	All	2.59	1.41	0.95	4.9e-13	3.2e-16	0.0002
	Normal	3.32	2.07	1.32	7.1e-12	2.2e-16	1.6e-05
	Malignant	2.42	1.76	1.28	0.0002	2.9e-07	0.0021
	P value (Normal,Malignant)	0.0003	0.032	0.8184	-	-	-
Maximum expression level (median)	All	0.0019	0.0075	0.0271	1.5e-09	2.2e-16	8.7e-11
	Normal	0.0017	0.0033	0.0116	0.0007	2.7e-14	9.4e-10
	Malignant	0.0020	0.0067	0.0269	2.3e-08	9.7e-14	4.1e-08
	P value (Normal,Malignant)	0.4786	0.0001	0.0042	-	-	-

^a^ The eight age groups were integrated into three as small number of newly birthed miRNAs has expression data, on which statistical analysis is likely to be spurious. Those miRNAs originated after the primate-rodent split was grouped into a group, referred as **Young**; those originated after rodent-non-mammalian split and before the primate-rodent split was grouped into **Middle**, and those originated before cow was grouped into **Old**. Wilcoxon statistics were used to test significances.

For each miRNA age groups, we next examined the differences in expression patterns between normal and malignant samples. The median of young miRNAs’ tissue specificity in cancer samples is significantly lower than that in normal samples; however, there is no distinct difference in tissue specificity for old miRNAs, which indicated that young miRNAs are expressed in more types of malignancies (i.e., “tissues”) than older miRNAs (Table 3). The median expression level of older miRNAs in malignant samples is significantly higher than in normal samples, and there is no distinct difference in the median expression level of young miRNAs between malignant and normal samples (Table 3).

To further estimate the involvement of miRNA expression tendency in malignant cells, we calculated the probability of finding a miRNA expressed in malignant samples using the hypergeometric test. Young miRNAs are more likely to be associated with malignancy, the median probability values decreasing with increasing miRNA age Additional file 1: Figure S6. Though young miRNAs are under stricter control than that of older miRNAs, a newly emerged miRNA might target mRNAs simply by chance, and that many of these interactions are likely to be deleterious. The data suggests that the regulation of young miRNAs might evolve in a way of trial and error to accommodate their biological function.

As expression patterns are very complex and highly dependent on experimental and biological conditions, an alternative expression dataset was further used for this analysis. To determine the tissue-specificity of human miRNAs, Hsu *et al.* detected the expression levels of 224 miRNAs in 18 major human normal tissues by using a real-time PCR-based method [39]. We got the same result, higher degree of tissue specificity and lower abundance for young miRNAs, when using this alternative data and when comparing two groups (young and old) (Additional file 1: Table S4).

## Discussion

The data showed that miRNA genes with new functional sequences accumulated more dynamically in vertebrates than that observed in Drosophila, which correlated with the increase in morphological complexity. It has been suggested that newly activated miRNAs may be part of general mechanism by which speciation occurs [18] and that acquisition of miRNA genes apparently speed up with evolution of organismal complexity [6,9]. Hence, analysis of miRNAs phylogenetics could be a useful starting point to explore the molecular basis of morphological complexity [6,9].

Such rapid emergence rate raised the question whether newly emerged miRNA genes have undergone rapid sequence evolution. We observed different levels of evolutionary selection over sequences of miRNA genes originated over a different time-scale. The data showed that relatively young miRNA genes may have undergone rapid sequence evolution when compared with old ones. A direct explanation may be that old miRNA genes have more important functions than new ones. Meanwhile, another interpretation should not be excluded, that is, a newly emerged miRNA may be evolving rapidly for the cooperation with its target transcripts before it is fully integrated into the regulatory network. The fact that miRNAs of different ages are often predicted/verified to target the same or overlapping sets of genes, emphasized our attention to those newly birthed miRNAs.

Regulation function of newly emerged “miRNAs”, when they find their targets, would probably be detrimental [18], however, they may also serve as substrate for natural selection of beneficial target interactions [13,19,20]. We observed higher degree of tissue specificity and lower expression level in young miRNAs, and different expression tendency between young and old miRNAs. The well-regulated expression of newly birthed miRNAs may reduce their effect on potential target mRNAs, consequently accommodate their relatively high level of sequence variation.

The present study has certain limitations that need to be taken into account, such as, some analysis may have bias towards well-studied miRNA genes or from the complex expression patterns of different miRNAs across multiple conditions. However, some of the limitations may leave clues for future research under the same theme.

## Conclusions

In this work, we thoroughly studied patterns of human miRNAs evolution over long evolutionary time. We divided the human known miRNA genes into eight age groups according to their time of origin. Our data showed that for miRNAs of different ages, evolution patterns are quite different in many aspects, from their precursor sequence and secondary structures (criteria of miRNAs identification), to their expression and targeting (the most important issues to elucidate miRNAs function). This study calls our attention to miRNAs’ origin time in the miRNAs functional analysis, in future studies.

## Methods

### Data

The sequence and annotation of human (hg18) and yeast (sacCer2) reference genomes were downloaded from UCSC Genome Browser database (http://hgdownload.cse.ucsc.edu/goldenPath/) [40]. Human SNPs and small insertions and deletions (indels) from dbSNP build 130 was downloaded from UCSC Genome Browser database (http://hgdownload.cse.ucsc.edu/goldenPath/) [40]. Human miRNA sequences and genomic coordinates were retrieved from the miRBase 15 release (http://www.mirbase.org/) [41]. Annotation of genomic position for all known miRNAs in 63 species were extracted from miRbase. We obtained a mammalian miRNA expression atlas of small RNA library sequencing from Landgraf *et al.*[38].

### Inference of human known miRNAs’ age

Phylogenetic analyses were carried out for ten species (human, chimp, orangutan, rhesus, mouse, rat, dog, horse, cow, chicken, and zebrafish). The annotation of miRNA genes remains difficult in some species, and there is a probability that some human miRNA genes might have not been identified in some other species yet. To address this problem, we mapped the sequences of human miRNA precursors to the genomes if the miRNA genes have not been annotated in that species, then we extracted the homologous sequences to examine whether they have miRNA features predicted by microPred [27]. We combined the annotations in miRBase [41] and the prediction results together to infer human miRNA genes’ ages. Members for each miRNA family were mapped to the phylogeny tree of the ten species. Human miRNAs’ ages were inferred by using maximum parsimony criterion, which requires that the miRNA family should have the least evolutionary changes (gains and losses) along the phylogeny tree. Human known miRNAs were thus classified into eight intervals (groups) based on their evolutionary ages estimated by maximum parsimony method.

### Birth-death rate inference of human known miRNAs

New miRNA genes face the pressure of decay after their fixation in the population, random mutations on a hairpin structure are known to have a strong tendency to destabilize it [42]. Lu *et al*. formulated the rate of decay as a survival function [24] (*i.e.*, the probability that a new miRNA survives to time t). The survivorship of putative functional miRNAs may be age dependent, as they may be longer-living due to selection against degenerative mutations. A gamma distribution was thus used to approximate the probability density of survivor for miRNA genes at time t.

A birth-and-death model was developed to account for the age distribution of the human miRNAs in the same way Lu *et al.* used [24], which incorporates a constant birth rate v and a survivorship function P(t). The survivorship function should satisfy P(0) = 1 and P(∞) = 0, which was modeled as a Gamma distribution function in this analysis. The number of preserved miRNAs within age interval [0, t] is then given by

$$N\left(t\right)\;=\;\upsilon \;\int_{0}^{t}P\left(t\right)\mathrm{dt}$$

which was fitted to the observed age distribution by the least square method. The numeric algorithm implemented by the ‘nlminb’ function in the R package was used to estimate the parameters in the model.

### Calculation of SNP/Indel density

Mature sequences and sequences outside mature regions of precursors were constructed respectively to account for the different structural and functional constraints, and the flanking regions around miRNAs were used as neutral background. SNP/Indel density was expressed as the averaged number of SNPs/Indels in the total sequence.

### Calculation of substitution rates

Multiple sequence alignments were constructed on the basis of the pairwise alignments, those homologous sequences without miRNA features were excluded. The alignments for precursors and mature sequences were constructed respectively to account for the different structural and functional constraints, and the flanking regions around miRNAs were used as neutral background. All miRNAs with the same age interval were concatenated together as individual miRNAs are too short for reliable statistical inference. Phylogenetic analyses of the concatenated sequences were analyzed by using the ‘baseml’ program in the PAML package [43]. The substitution model used in the simulation is K80, and the molecular clock was assumed to tick at a constant rate. The substitution rate was estimated as the height of the tree divided by the divergence time.

### Analysis of pre-miRNAs’ features

RNAfold [44] was used to calculated minimal free energy of human pre-miRNAs’ secondary structure. Since minimal free energy (MFE) will be influenced by the pre-miRNA length, a normalized MFE (NMFE) was calculated as MFE divided by length. Randfold [45] was used to calculate the probability that a pre-miRNA structure has an MFE that is lower than a randomly shuffled sequence. MicroPred program [27] was used to calculate 48 features of human pre-miRNAs. For each human known miRNA age groups, average was plotted in the figure with error bars of standard deviation. Mirident [29] was used for identification of s-motifs.

### Expression analysis of human known miRNAs

MicroRNA abundance was normalized to the counting rate for the case of total clone count of all miRNAs in a sample. To avoid variability in miRNAs expression patterns between different experimental or biological conditions, the maximum abundance among the small RNA libraries was used to measure miRNAs’ expression level [16]. We calculated the expression specificity of a miRNA according to the information content [38],

$$\log_{2}\;n\;+\;\sum_{i=1}^{n}Pi^{\log_{2}}\mathrm{Pi}$$

where *n* is the number of major tissues, and *p*_*i*_ is the percentage of expression abundance in major tissue *i*.

To estimate the expression tendency in normal or malignant samples, we calculated the probability of finding a miRNA in malignant samples using hypergeometric test.

### Prediction of miRNAs’ target genes

Two sets of data were used for the analysis of miRNAs’ targets, *i.e.*, target dataset predicted by TargetScan program [46] and experimentally verified target dataset from Hafner *et al.*[37]. MicroRNA target prediction was done by using TargetScan program [46]. As conserved pairing to the seed region can be sufficient on its own for predicting conserved targets above the noise of false-positive predictions, we further screened predicted hits for those conserved canonical 7–8 nt seed-matched sites, which are more likely to yield higher signal above background [47].

### Functional enrichment of human genic miRNAs’ host genes

As coding-gene annotation is currently unavailable for orangutan and rhesus, we classified human host genes into six intervals(groups) based on their evolutionary ages following the same method used for miRNAs. Gene Ontology (GO) analysis was performed using DAVID (http://david.abcc.ncifcrf.gov) [48], by examining the molecular function in which genes hosting miRNA(s) are involved, using Ensembl Gene IDs and the entire human genome as a background model. P-values were calculated using Fisher Exact statistics.

## Competing interests

The authors declare no conflict of interest.

## Authors’ contributions

YZ, HS and QN carried out the study. ZW, BL and SY participated the study. HS and GS wrote the manuscript. YL designed and sponsored the study. All authors read and approved the manuscript.

## Supplementary Material

Additional file 1 Supplementary materials. Click here for file
