# Supplementary material for: Evolutionary relationships between miRNA genes and their activity
Source: BMC Genomics. 2012 Dec 22;13:718. doi: 10.1186/1471-2164-13-718 (PMC3544654; doi:10.1186/1471-2164-13-718)
Supplement: Additional file 1 — Supplementary materials. [file 1471-2164-13-718-S1.pdf]

# Supplementary materials

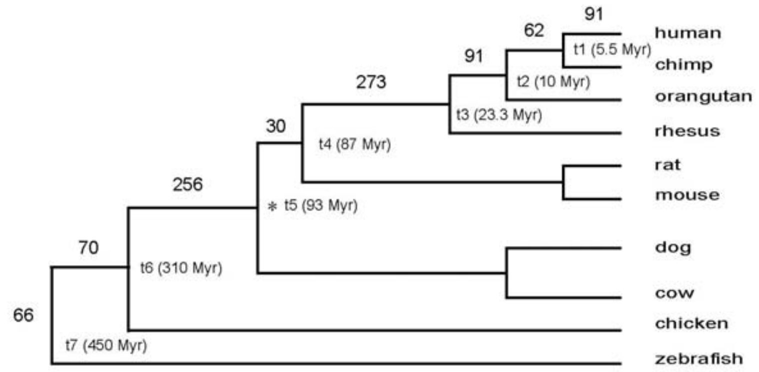


**Figure S1**: Accumulation of human miRNAs during evolution.

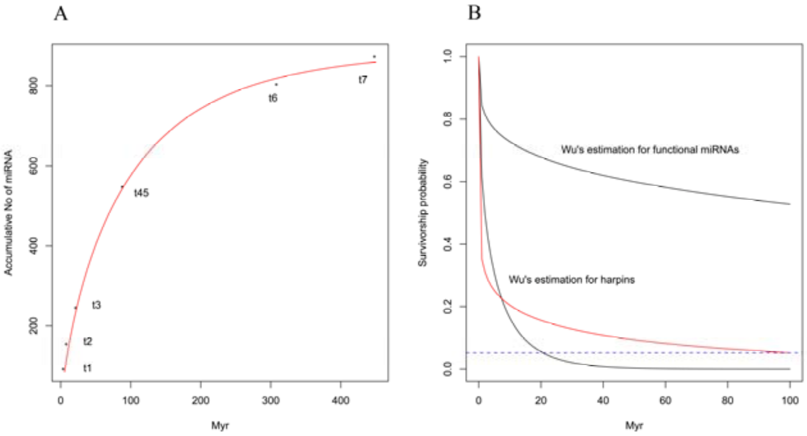


**Figure S2:** (A) Number of human miRNAs originated at each time point. (B) Survival probability of human miRNA (shape =0.51, rate=0.007).

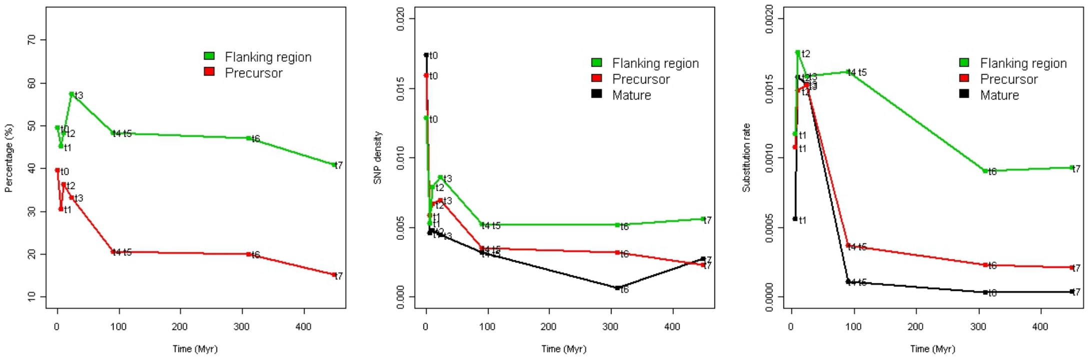


**Figure S3**: Evolutionary mutations for each group of human known miRNAs. (A) Percentage of miRNAs containing SNP(s). (B) SNP density. (C) Substitution rates.

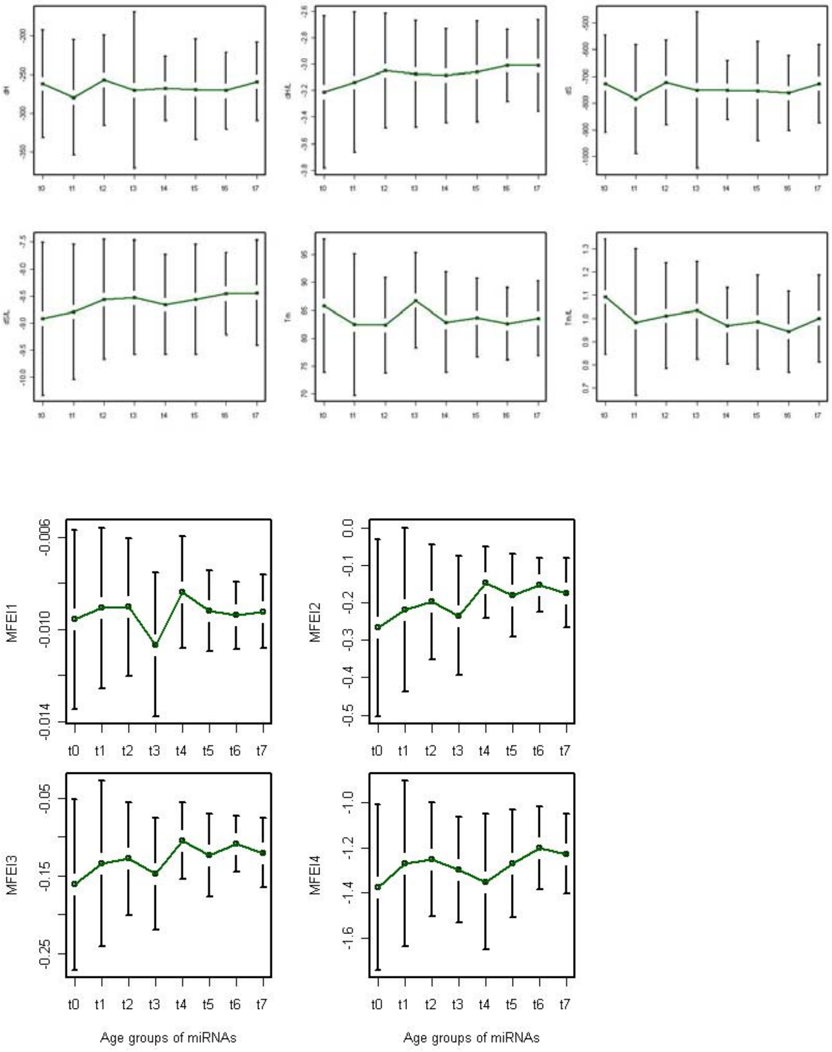


**Figure S4**: Relationship between miRNA evolutionary age and pre-miRNA features

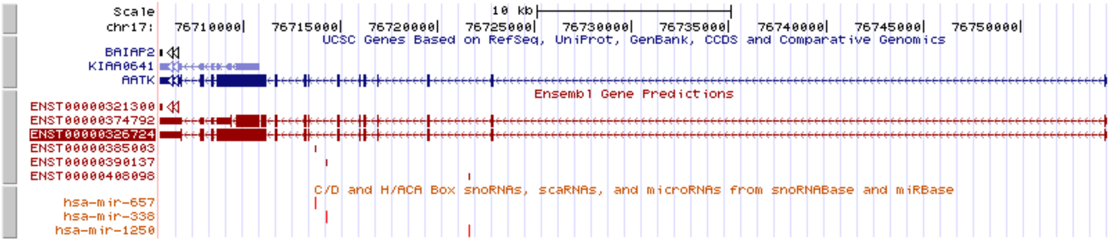


**Figure S5**: MicroRNA genes with distinct origin encoded by the introns of AATYK gene

**Table S1**: Genomic contexts of human known miRNAs of each age groups

| | Genic | | Intergenic | |
|---|---|---|---|---|
| Age group | Number | Percentage (%) | Number | Percentage (%) |
| t0 | 60 | 66 | 31 | 34 |
| t1 | 47 | 76 | 15 | 24 |
| t2 | 55 | 60 | 36 | 40 |
| t3 | 169 | 62 | 104 | 38 |
| t4 | 24 | 80 | 6 | 20 |
| t5 | 139 | 54 | 117 | 46 |
| t6 | 48 | 69 | 22 | 31 |
| t7 | 46 | 70 | 20 | 30 |

The chi-square test of independence was used to test whether the observed frequencies differ significantly from the expected. We got a p value of 0.2289.

**Table S2:**

A. Number of target genes predicted by TargetScan program [1].

| | Age group | t0 | t1 | t2 | t3 | t4 | t5 | t6 | t7 |
|---|---|---|---|---|---|---|---|---|---|
| Conserved targets[a] (>= 2) | Median | 236 | 293 | 282 | 284 | 349 | 311 | 452 | 485 |
| | Mean | 319 | 376 | 333 | 398 | 356 | 401 | 492 | 552 |

**B.** P values were got by pair-wise comparisons using wilcoxon statistics.

| Age group | t0 | t1 | t2 | t3 | t4 | t5 | t6 | t7 |
|---|---|---|---|---|---|---|---|---|
| t0 | - | 0.2043 | 0.3860 | 0.1222 | 0.1729 | 0.0310 | 0.0013 | 7.3e-05 |
| t1 | | - | 0.5011 | 0.9355 | 0.6438 | 0.6486 | 0.0647 | 0.0074 |
| t2 | | | - | 0.4403 | 0.2825 | 0.1526 | 0.0055 | 0.0004 |
| t3 | | | | - | 0.6562 | 0.4365 | 0.0209 | 0.0007 |
| t4 | | | | | - | 0.9404 | 0.1383 | 0.0312 |
| t5 | | | | | | - | 0.0500 | 0.0026 |
| t6 | | | | | | | - | 0.3424 |

[a] TargetScan was used to search annotated human 3' UTRs for the presence of conserved 8mer and 7mer sites that match the seed region of each miRNA.

**Table S3**:

**A.** Number of target genes experimentally verified [2].

| Age groups [a] | Young | Middle | Old |
|---|---|---|---|
| Median | 3 | 119 | 156 |
| Mean | 6 | 310 | 320 |

**B.** P values were got by pair-wise comparisons using wilcoxon statistics.

| | Young | Middle | Old |
|---|---|---|---|
| Young | - | 0.0080 | 0.0008 |
| Middle | | - | 0.3288 |

[a] The eight age groups were integrated into three as the same way in table 3, as small number of newly birthed miRNAs has experimentally verified data.

**Table S4**: Abundance and tissue specificity of human known miRNAs' expression.

| Data sources | Measures | Samples | Young[a] | Old[a] | P value (Young, Old) |
|---|---|---|---|---|---|
| Data from Landraf et al. [3] | Tissue specificity score (median) | All | 2.42 | 1.23 | 1.4e-15 |
| | | Normal | 3.32 | 1.73 | 2.2e-16 |
| | | Malignant | 2.34 | 1.54 | 6.7e-05 |
| | | P value (Normal, Malignant) | 1.6e-05 | 0.2855 | |
| | Maximum expression level (median) | All | 0.0027 | 0.0121 | 3.4e-15 |
| | | Normal | 0.0019 | 0.0058 | 3.6e-07 |
| | | Malignant | 0.0017 | 0.0109 | 2.2e-10 |
| | | P value (Normal, Malignant) | 0.9107 | 2.4e-05 | |
| Q-PCR miRNA profiling [4] | Tissue specificity score (median) | Normal | 3.86 | 0.45 | 5.4e-11 |
| | Maximum expression level (median) | Normal | 170200 | 328500 | 0.0331 |

[a]The eight age groups were integrated into two as small number of newly birthed miRNAs has expression data, on which statistical analysis is likely to be spurious. Those miRNAs originated after the rodent was grouped into a group, referred as Young, and those originated before rodent was grouped into Old. Wilcoxon statistics were used to test the significances.

## References:


1. Lewis BP, Burge CB, Bartel DP: **Conserved Seed Pairing, Often Flanked by Adenosines, Indicates that Thousands of Human Genes are MicroRNA Targets**. *Cell* 2005, **120**(1):15-20.
2. Hafner M, Landthaler M, Burger L, Khorshid M, Hausser J, Berninger P, Rothballer A, Ascano M, Jr., Jungkamp AC, Munschauer M *et al*: **Transcriptome-wide identification of RNA-binding protein and microRNA target sites by PAR-CLIP**. *Cell* 2010, **141**(1):129-141.
3. Landgraf P, Rusu M, Sheridan R, Sewer A, Iovino N, Aravin A, Pfeffer S, Rice A, Kamphorst AO, Landthaler M *et al*: **A mammalian microRNA expression atlas based on small RNA library sequencing**. *Cell* 2007, **129**(7):1401-1414.
4. Hsu SD, Chu CH, Tsou AP, Chen SJ, Chen HC, Hsu PW, Wong YH, Chen YH, Chen GH, Huang HD: **miRNAMap 2.0: genomic maps of microRNAs in metazoan genomes**. *Nucleic Acids Res* 2008, **36**(Database issue):D165-169.
